# The relationship between demographic factors and known risk factors with breast cancer in women aged 30–69

**DOI:** 10.1097/MS9.0000000000002114

**Published:** 2024-05-06

**Authors:** Mohammad-Ali Jahani, Behnaz Ghasemi, Seyed Amir Soltani, Malihe Naderi, Hossein-Ali Nikbakht, Seyedeh Nikoo Hashemi, Jamshid Yazdani Charati, Ghahraman Mahmoudi

**Affiliations:** a Social Determinants of Health Research Center, Health Research Institute, Babol University of Medical Sciences, Babol; b Hospital Administration Research Center, Sari Branch, Islamic Azad University; c Department of Biostatistics and Epidemiology, School of Health, Health Sciences Research Center, Addiction Institute, Mazandaran University of Medical Sciences, Sari; d Department of Microbiology and Microbial Biotechnology, Faculty of Life Sciences and Biotechnology, Shahid Beheshti University, Tehran; e Doctorate of Medicine, Shiraz University of Medical Sciences, Shiraz; f Golestan University of Medical Sciences, Gorgan, Islamic Republic of Iran

**Keywords:** breast cancer, risk factors, women’s health

## Abstract

**Background:**

Breast cancer is one of the most important causes of cancer deaths in women. The present study was conducted to determine the relationship between demographic factors and known risk factors with breast cancer in women aged 30–69.

**Method:**

This case–control study was conducted with two matched and unmatched control groups. Three hundred fifty women aged 30–69 with breast cancer, 350 age-matched women without cancer, and 350 not age-matched women were included in the study. Controls were selected from the records of women whose breast cancer screening results were normal. Study subjects were evaluated regarding the risk factors for breast cancer. The data collection tool was a checklist including the risk factors investigated in the integrated health system. The collected data were analyzed utilizing SPSS22 software at a significance level of less than 0.05.

**Results:**

The average age in the case group was 46.63±11.77 years and 49.61±8.39 in the unmatched control group. The average age of marriage in the case group was 21.54±4.31, and the average age of women at first pregnancy in the case group was 24.06±3.39 years. In the case group, 163 people (46.57%) lived in the city, 221 people (63.14%) were over 40 years old, and 337 people (96.28%) were married. In multivariate analysis, the variable ‘age of marriage’ 0.821 (0.691–0.976) and ‘age of first pregnancy’ 1.213 (1.020–1.443) showed a significant relationship with breast cancer which were observed as predictors of breast cancer in comparison to the unmatched control group (*P*-value <0.05).

**Conclusion:**

The age of the first pregnancy and the type of delivery were observed as predictors of breast cancer. Therefore, by performing breast cancer screening in women who are exposed to these risk factors, early diagnosis of the disease and increasing the speed of their treatment can be significantly helped.

## Introduction

Highlights

- The average age of marriage in the case group was 21.54±4.31, and the average age of women at first pregnancy in the case group was 24.06±3.39 years.
- The variable ‘age of marriage’ 0.821 (0.691–0.976) and ‘age of first pregnancy’ 1.213 (1.020–1.443) showed a significant relationship with breast cancer which were observed as predictors of breast cancer in comparison to the unmatched control group.
- The age of the first pregnancy and the type of delivery were observed as predictors of breast cancer.

Death rates due to breast cancer have declined in most European countries over the past 20 years^1,2^ due to early detection and improved treatments. Mammography screening has reduced breast cancer deaths by about 20%^3^. In the United States, approximately one in every eight women develop breast cancer, which is the second leading cause of cancer death in women^4,5^; breast cancer is more common in the white population^6^. During the past years, breast cancer has surpassed lung cancer as the most common fatal cancer, and breast cancer in women is becoming the leading cause of death from malignancies with a 6.9% rate^7^. There are groups of women who are considered at risk of developing breast cancer; however, most patients are not aware of their risk status^8,9^. According to studies, the average age of people with breast cancer in Iran is 5–10 years younger than the world average, so one out of every 7–8 women has the risk of developing breast cancer^10^. According to recent statistics, the death rate due to breast cancer in Iran has more than doubled^11,12^. The incidence of various cancers, especially breast cancer, has been increasing and incurs a lot of human and financial costs to society, the health system, and the people. Biennial screening mammography is recommended for women aged 50–74 at no particular risk in France. However, the risk of cancer among moderate-risk women varies according to known risk factors, such as breast density and family history of breast or ovarian cancer^13–15^.

Today, risk assessment is considered one of the appropriate ways to estimate, evaluate, prevent, and control the probability of noncommunicable diseases, so a tool is designed that examines several risk factors such as age, sex, blood pressure, smoking, and other relatable factors to determine the probability of the disease in question and act to reduce it^16–18^. In women, a personalized approach to prevention and early detection based not only on age but also on different risk factors is a promising strategy, as it should adapt screening intervals and methods according to individual risk, leading to the detection of breast cancer in the early and treatable stages^13^.

Studies have been carried out in the field of cancer risk assessment; Momayezi *et al*.^19^ showed in their research that breastfeeding for less than 11 months, hormone therapy after menopause, the age of onset of menopause after 46 years, and the use of birth control pills were among the significant risk factors for breast cancer. Mousavizadeh *et al*.^20^ showed that the age of the first pregnancy is also one of the main risk factors with high strength for breast cancer development in the region. The current knowledge of the etiology of cancer does not have a good perspective regarding primary prevention. Still, the goal should be based on primary prevention, eliminating risk factors, and improving the level of education about cancer. Screening for cancer, which is a type of secondary prevention, leads to early detection of cancer, which in turn is effective in better treatment and reducing mortality^21^. Daly *et al*. showed in their research that breast cancer is the most common malignancy among women under 40 years of age, and the rate of breast cancer has increased steadily in the last 20 years. Among risk factors, physical activity, alcohol consumption, smoking, and eating habits are the modifiable ones, and of course, there are risk factors such as age, sex, and family history, which are nonmodifiable factors that need to be addressed for early detection of cancer^22^.

Considering that some of the risk factors of breast cancer cannot be changed easily, its prevention is directed towards factors that are subject to change. This information shows the need for preventive strategies in the mentioned field. By examining risk factors such as age, activity level, smoking, and other relatable factors, it is possible to determine the probability of cancer occurrence, help the early detection of the disease, and increase the speed of treatment^6^. For this reason, it is necessary to conduct research to obtain the pattern of occurrence and distribution of cancers, especially breast cancer, which depends on regional conditions, nutritional habits, and genetic factors to identify risk factors regionally. Therefore, it is crucial to take appropriate steps through planning by the authorities to reduce the incidence of breast cancer, as well as early diagnosis and improving the effective treatment of this disease. So, the purpose of this research is to investigate factors such as the amount of smoking, alcohol consumption, drug use, age distribution, the presence of benign breast cysts, breast biopsy status, physical activity, family history of breast and ovarian cancer, BMI, blood pressure, diabetes, nutrition pattern, vitamin D supplement use, history of pregnancy and childbirth, and history of breastfeeding in affected and healthy women. These risk factors have been investigated comparatively in healthy and affected females; using the results of this research, we are able to provide more comprehensive and complete information to recognize the risk factors and help reduce the incidence and mortality of breast cancer in women aged 30–69.

## Method

The present study is an applied quantitative sibling case–control study conducted in 2021. The study population was the electronic records of 303 642 women aged 30–69 who lived and who have been covered by primary health care and their information has been registered in the Integrated Health System (IHS).

After the proposal was approved by the university’s research council and after obtaining the ethics code number. We presented it to University of Medical Sciences, and in coordination with senior officials of University of Medical Sciences, a permit was obtained to access the information of women aged 30–69. Data including age, age of marriage, age of first pregnancy, age of last pregnancy, breastfeeding, type of delivery, history of hypertension, diabetes, hyperlipidemia, vitamin D supplement use, smoking, drugs, and alcohol were extracted from http://sib.arums.ac.ir/ and entered in the Excel software. Ethical considerations regarding maintaining the confidentiality of the information and the impartiality of the researcher were emphasized in all stages of the research.

In the current study, one case group and two control groups were used; the case group includes all the electronic records of women aged 30–69 who, according to the registered information, had abnormal breast cancer screening results, and the person was confirmed to have breast cancer. Their data has been entered in the cancer registration system as verified breast cancer patients, which included 350 cases (all cases of breast cancer patients). The control group included all the electronic records of women aged 30–69 who, according to the IHS, had normal breast cancer screening results and were not confirmed to have breast cancer, and were evaluated as healthy in terms of breast cancer. 159 417 subjects had a normal screening result.

To increase the study’s validity, the size of each control group was chosen to be the same size as the case group (350 subjects), so the control sample size was 700 cases, which were examined in two groups of 350 people. In one control group, age matching was done with the case group. Random sampling was implemented to determine the size of the matched control group. The entire electronic files of women aged 30–69 (healthy in terms of breast cancer screening) were obtained from the IHS, which included 159 417 files. The first case was randomly selected and included in the age-matched control group. A regular random sampling method was used to select the group members in the unmatched control group. So, the total number of women’s files evaluated for breast cancer screening was 159 417, numbered from 1 to the last. Considering that the sample size should have been 350, the number k=N/n=159 417/350=455 was obtained according to the formula. Between the numbers 1 and 455, a number was chosen by lottery, and the number 78 was obtained; this number was related to the number of the first sample. Then, we obtained the file numbers in an Excel file using the formula method. We added 455 to the first number and got the second number. In the same way, the total number of samples reached 350, and the size of the control sample 2 was selected; if the selected case was repeated and was established in the control group 1, to avoid repetition, that case was removed, and the next case was replaced. The work has been reported in line with the strengthening the reporting of cohort, cross-sectional and case–control studies in surgery (STROCSS) criteria^23^.

The inclusion criteria in this study included women aged 30–69, living in between 2016 and 2021, and having an active health record in primary health care centers. Women under 30 and over 70 and those with incomplete information and health records were excluded from the study. In the next step, the data collected in Excel was entered into SPSS22 software. Statistical analysis was carried out using independent samples *t*-test (matching was done in groups), Fisher’s exact test, *χ* ^2^ test, and univariate and multivariate logistic regression with backward elimination method test at a significance level of less than 0.05.

### Ethical approval

This study was done after holding the ethical code registered data were used. All methods were performed in accordance with the relevant guidelines and regulations of the Declaration of Helsinki.

## Results

The research findings showed that the average age in the matched case and control groups was 46.63±11.77 years, and in the nonmatched control group, it was 49.61±8.39. The average age of marriage in the case group was 21.54±4.31, and the average age of the woman in the first pregnancy in the case group was 24.06±3.39 years. In the case group, 163 people (46.57%) lived in the city, 221 people (63.14%) were over 40 years old, and 337 people (96.28%) were married (Table 1).

**Table 1:** Demographic information and some variables of the studied subjects.

		‘Investigated group’ Variable mean (Frequency (%))	
Variable	Case	Matched control	Unmatched control
Place of residence			
Urban	163 (46.57%)	158 (45.14%)	224 (64%)
Rural	187 (53.42%)	192 (54.85%)	126 (36%)
Age group (Years)			
<29	00	00	0 (0.00)
30–39.9	129 (36.85%)	129 (36.85%)	49 (14%)
40–49.9	78 (22.28%)	78 (22.28%)	137 (39.14%)
50–59.9	78 (22.28%)	78 (22.28%)	114 (32.57%)
>60	65 (18.57%)	65 (18.57%)	50 (14.28%)
Marital status			
Single	4 (1.14%)	2 (0.57%)	15 (4.28%)
Married	337 (96.28%)	337 (96.28%)	313 (89.42%)
Widow	7 (2%)	6 (1.71%)	12 (3.42%)
Divorced	2 (0.57%)	5 (1.42%)	10 (2.85%)
Variable			
—	Mean±SD		
Age			
—	46.63±11.77	46.63±11.77	49.61±8.39
Age at marriage			
—	21.54±4.31	21.89±3.28	23.44±4.63
Age at when spouse’s death			
—	42±14.45	41.09±6.13	46.25±11.64
Number of children			
—	3.04±1.41	3.47±1.46	3.02±1.42
Age at first pregnancy			
—	24.06±3.39	23.81±3.30	25.75±4.77
Age at latest pregnancy			
—	32.80±4.69	33.21±4.48	34.86±4.26
Number of months breastfeeding the last child			
—	11.53±0.62	11.58±0.59	11.74±1.14

Twenty people (5.71%) in the case group had total cholesterol of more than 200, 42 people (12.00%) had prehypertension, and 48 people (13.71%) had blood pressure above 140/80.

Regarding diabetes, in the case group, 13 people (71.3%) had prediabetes, four people (1.14%) had gestational diabetes, and 49 people (14.00%) were cancer patients with diabetes and had fasting blood glucose levels of more than 126 mg/dl.

Family history of breast cancer in first-degree relatives was also reported in 79 people (22.57%) of cases (Table 2).

**Table 2:** Information on the disease and health status of the studied subjects.

	Investigated group		
Variable	Case	Matched control	Unmatched control
Blood Cholesterol (>200)			
Yes	20 (5.71%)	11 (3.14%)	7 (2%)
No	330 (94.28%)	339 (96.85%)	343 (98%)
High blood pressure(≥90/140 mmHg)			
Healthy	260 (72.28%)	315 (90%)	325 (92.85%)
Prehigh blood pressure	42 (12%)	12 (3.42%)	10 (2.85%)
High blood pressure	48 (13.71%)	23 (6.57%)	15 (4.28%)
Diabetes (≥126 mg/dl)			
Healthy	284 (81.14%)	331 (94.57%)	336 (96%)
Prediabetes	13 (3.71%)	2 (0.57%)	3 (0.85%)
Diabetes	49 (14%)	15 (4.28%)	11 (3.14%)
Gestational diabetes mellitus	4 (1.14%)	2 (0.57%)	0 (0%)
Family history of cancer in first-degree female relatives			
Yes	79 (22.57%)	7 (2%)	4 (1.14%)
No	271 (77.42%)	343 (98%)	346 (98.85%)
BMI			
Underweight	34 (9.71%)	30 (8.57%)	27 (7.71%)
Normal weight	102 (29.14%)	232 (66.28%)	205 (58.57%)
Overweight	120 (34.28%)	64 (18.28%)	95 (27.14%)
Obesity class 1	6 (22%)	11 (3.14%)	18 (5.14%)
Obesity class 2	11 (3.14%)	8 (2.28%)	5 (1.42%)
Obesity class 3	6 (1.71%)	2 (0.57%)	0 (0%)
Type of delivery			
Cesarean section	49 (14.24%)	31 (8.90%)	117 (35.77%)
Normal	295 (85.75%)	317 (91.09%)	210 (64.22%)
Age of the woman at the first pregnancy			
-	24.06 ± 3.39	23.81 ± 3.30	25.75 ± 4.77
Age of the woman at the last pregnancy			
-	32.80 ± 4.69	33.21 ± 4.48	34.86 ± 4.26
Breastfeeding with maternal milk in the last child			
Yes	312 (90.69%)	323 (93.28%)	330 (94.28%)
No	32 (9.3%)	25 (7.14%)	20 (5.71%)
Physical activity status			
Mild	304 (86.85%)	277 (79.14%)	245 (70%)
Moderate	46 (13.14%)	71 (20.28%)	98 (28%)
Severe	0 (0%)	2 (0.57%)	7 (2%)
Vitamin D consumption			
Yes	294 (84%)	339 (96.85%)	340 (97.14%)
No	56 (16%)	11 (3.14%)	10 (2.85%)
Existence of cyst			
Yes	34 (9.71%)	0 (0%)	0 (0%)
No	316 (90.29%)	350 (100%)	350 (100%)
Breast sampling history			
Yes	55 (15.27%)	0 (0%)	0 (0%)
No	295 (84.28%)	350 (100%)	350 (100%)

Table 3 shows the relationship of the investigated variables with breast cancer based on the results of univariate and multivariate logistic regression tests in matched case and control groups. The table below shows a significant relationship between the two variables when the *P*-value is <0.05 or the number 1 is not within the 95% CI. Considering that in the multivariable logistic regression test, the *P*-value of the Hosmer and Lemeshow Test was greater than 0.05, the model has the necessary adequacy (Hosmer and Lemeshow Test *P*-value=0.307).

**Table 3:** Determinants of breast cancer based on univariate and multivariate logistic regression analysis (compared to controls matched with patients).

		Univariate analysis		Multivariate analysis	
Variable	Number (*N*)	OR (95% CI)	*P*	OR (95% CI)	*P*
Age	700	0.972 (0.958–0.986)	<0.001	0.984 (0.966– 1.003)	0.361
Age at marriage	694	1 (0.956–1.045)	0.998	0.821 (0.691–0.976)	0.026
Age at first pregnancy	692	1.023 (0.978–1.070)	0.319	1.213 (1.020–1.443)	0.029
Age at last pregnancy	692	0.981 (0.949–1.013)	0.246	1.034 (0.802–1.143)	0.829
Place of residence					
City	321	01.065 (0.791–1.434)	0.679	0.369 (0.265–1.106)	0.078
Village	378	Reference group			
Marital status					
Married	674	1 (0.457–2.189)	1	1.078 (0.812–1.162)	0.386
Other (Single/Divorced/Widow)	26	Reference group			
Type of delivery					
Cesarean section	80	1.699 (1.054–2.737)	0.029	1.012 (0.902–1.243)	0.150
Natural	612	Reference group			
Breastfeeding					
Does not have	51	1.586 (0.888–2.831)	0.119	1.130 (0.602–1.298)	0.141
Has	614	Reference group			
Positive family history of cancer					
Yes	11	0.566 (0.164–1.953)	0.368	0.750 (0.706–1.233)	NS*
No	689	Reference group			
High blood cholesterol					
Yes	31	1.135 (1.024–2.45)	0.045	1.330 (0.980–1.845)	0.086
No	669	Reference group			
High blood pressure					
Prehypertension/High blood pressure	125	1.251 (1.154–2.312)	0.038	1.072 (0.774–1.206)	0.071
Healthy	575	Reference group			
Diabetes					
Prediabetes/Diabetes/ Gestational diabetes	85	1.653 (1.114–2.020)	0.015	1.822 (0.994–2.161)	0.113
Healthy	615	Reference group			
Vitamin D consumption					
Yes	633	0.798 (0.655–1.648)	0.671	0.926 (0.879–1.066)	NS
No	67	Reference group			
Physical activity					
Moderate/Intense	119	0.908 (0.875–2.301)	0.461	0.978 (0.974–2.404)	NS
Light	581	Reference group			
BMI					
Thin	57	Reference group			
Normal	437	0.822 (0.374–1.806)	0.625	0.964 (0.532–1.106	NS
Overweight	159	0.807 (0.433–1.501)	0.498	1.022 (0.374–1.806)	NS
Obese	44	1.355 (0.693–2.652)	0.375	1.432 (0.374–1.806	NS

* NS (nonsignificant) these variables were removed by applying multivariate logistic regression with backward elimination method.

In the comparison of the case group with the matched control group in univariate analysis, only the variables of age 0.972 (0.958–0.986), type of delivery 1.699 (1.054–2.737), high cholesterol 1.135 (1.024–2.45), high blood pressure 1.251 (1.154–2.312), and diabetes 1.653 (1.114–2.020) showed a significant relationship with cancer (*P*-value <0.05) and no significant relationship with breast cancer was observed in other variables. Multivariate analysis showed a significant relationship between the variables of ‘age of marriage’ and ‘age of first pregnancy’ with breast cancer, which were observed as predictors of breast cancer in the comparison of the case group with the matched control group (*P*-value <0.05). A significant relationship between breast cancer and the age of marriage was reported at 0.821 (0.691–0.976), so with an increase of one year in the age of marriage, the risk of breast cancer decreases by 17%, which can indicate the patients under study marrying at a young age. Regarding the age at first pregnancy, we identified a significant relationship with breast cancer 1.213 (1.020–1.443), so that with an increase of 1-year in the age of the first pregnancy, the risk of breast cancer increases by 21% (Table 3).

Table 4 shows the relation of the examined variables with breast cancer based on univariate and multivariate analysis in the case and unmatched control groups.

**Table 4:** Determinants of breast cancer based on univariate and multivariate logistic regression analysis (comparing unmatched controls with patients).

		Univariate analysis		Multivariate analysis	
Variable	Number (*N*)	OR (95% CI)	*P*	OR (95% CI)	*P*
Age	700	0.975 (0.958–0.986)	<0.001	0.967 (0.902–1.036)	0.070
Age at marriage	685	1 (0.966–1.045)	0.997	0.821 (0.691–0.976)	0.026
Age at first pregnancy	671	1.048 (0.978–1.070)	0.317	1.261 (1.028–1.513)	0.029
Age at last pregnancy	671	0.981 (0.849–1.022)	0.251	1.011 (0.871–1.174)	NS*
Place of residence					
City	387	1.152 (0.861–1.541)	0.654	1.040 (0.505–1.109)	0.074
Village	312	Reference group			
Marital status					
Married	654	1 (0.486–2.219)	1	1.056 (0.773–1.220)	NS
Other (Single/Divorced/Widow)	46	Reference group			
Type of delivery					
Cesarean section	166	1.712 (1.059–2.811)	0.022	1.081 (0.854–1.154)	0.150
Natural	505	Reference group			
Breastfeeding					
Does not have	52	1.461 (0.825–1.711)	0.112	1.229 (0.524–1.400)	0.102
Has	625	Reference group			
Positive family history of cancer					
Yes	83	0.766 (0.153–1.623)	0.245	0.989 (0.854–1.154)	0.150
No	613	Reference group			
High blood cholesterol					
Yes	27	1.150 (1.024–2.45)	0.046	1.309 (0.966–1.974)	0.098
No	673	Reference group			
High blood pressure					
Prehypertension/High blood pressure	115	1.272 (0.661–2.302)	0.040	1.089 (0.756–1.194)	0.076
Healthy	585	Reference group			
Diabetes					
Prediabetes /Diabetes/ Gestational diabetes	80	1.651 (1.122–2.026)	0.016	1.109 (0.989–2.154)	0.071
Healthy	620	Reference group			
Vitamin D consumption					
Yes	634	0.798 (0.655–1.648)	0.670	1.010 (0.791–1.048)	Ns
No	66	Reference group			
Physical activity					
Moderate/Intense	151	0.919 (0.851–2.278)	0.449	0.965 (0.809–1.954)	Ns
Light	549	Reference group			
BMI					
Thin	61	Reference group			
Normal	307	0.829 (0.421–1.208)	0.712	0.990 (0.510–1.090)	Ns
Overweight	215	0.816 (0.523–1.413)	0.518	1.689 (0.854–1.154)	Ns
Obese	117	1.451 (0.712–2.623)	0.345	1.336 (0.884–1.904	Ns

* NS (nonsignificant) these variables were removed by applying multivariate logistic regression with backward elimination method.

In the comparison of the case group with the unmatched control group in the univariate analysis, as in the matched control group, the variables of age 0.975 (0.958–0.986), delivery type 1.712 (1.059–2.811), high cholesterol 1.150 (1.024–2.45), high blood pressure 1.272 (0.661–0.302), and diabetes 1.651 (1.122–2.026) showed a significant relationship with cancer (*P*-value <0.05). In multivariate analysis, the variables age of marriage 0.821 (0.691–0.976) and age of first pregnancy 1.261 (1.028–1.513) showed a significant relationship with breast cancer. Compared with the control group, they were observed as predictors of breast cancer (*P*-value <0.05). With an increase of 1-year in the age of marriage, the risk of breast cancer decreases by 17%, which can indicate the marriage of the studied patients at a young age. Also, with an increase of one year in the age of the first pregnancy, the risk of breast cancer increases by 26%.

## Discussion

In comparing the case group with the matched control group by univariate analysis, as in the nonmatched control group, only the variables of age, type of delivery, high cholesterol, high blood pressure, and diabetes showed a significant relationship with cancer, and no significant relationship with other variables was observed, in multivariate analysis, the age of marriage and first pregnancy showed a significant relationship with breast cancer, which were observed as predictors of breast cancer in the comparison of the case group with the unmatched control group.

Breast cancer risk factors are divided into two categories: nonmodifiable risk factors that include age, sex, family history, genetic mutations, age of menstruation less than 11 years, age of menopause over 54 years, racial and ethnic background, status, high socioeconomic status, and history of some benign breast diseases. Modifiable risk factors include BMI above 30, alcohol consumption, old age at the time of first delivery, history of exposure to radiation, use of oral contraceptive pills, hormone replacement therapy after menopause, and high consumption of saturated fats. Adequate knowledge and understanding of the risk factors of breast cancer can play a significant role in prevention and, as a result, reduce the rate of this cancer among women in this province, so they were investigated in this study. Obesity (BMI of ≥ 30.0) has been associated with worse breast cancer survival in a meta-analysis and systematic review^24^. In another review, obesity was associated with worse breast cancer prognosis for women of all ages^25^. For T2DM, a retrospective study of breast cancer patients found that diabetes was independently associated with poorer breast cancer prognosis^26^. In a population-based study, breast cancer-specific mortality was higher among women with diabetes compared to nondiabetic patients^27^. The relationship between mammographic density and breast cancer survival has been studied in several cohort studies, but results have been inconclusive^28,29^. For other factors such as physical activity, the evidence is also not clear: in an RCT with an 8-year follow-up, no significant difference in disease-free survival was found between an exercise group and a usual care group^30^. To date, there is no evidence for an association between height or postdiagnosis alcohol consumption and breast cancer survival^31^. It has been shown in various studies that smoking and being exposed to cigarette smoke on a chronic basis have a significant effect on increasing the risk of breast cancer^32–34^, but in the present study, none of the groups reported smoking, using drugs and alcoholic beverages, which may be due to geographic conditions, genetics, quality of life, nutrition, and failure to provide correct information to health workers due to fear of judgment and concealment and other sociological factors, so a statistical test was not performed for these variables.

Age distribution is one of the most important risk factors for new cases of breast cancer, and in the present study, a significant relationship between age and the increase in the risk of breast cancer was observed, which is in line with the previous research. About 50% of breast cancers occur in women 65 years and older and 35% after 90 years^35,36^. Age is considered a major factor in identifying and treating patients^37^. There is a lot of evidence that several nongenetic risk factors are associated with developing breast cancer^38^, and breast cancer is a hormone-related disease, so the effect of different factors is not the same between premenopausal and postmenopausal patients^39^ and the onset of menstruation at an older age in women with a positive family history has a strong protective effect against breast cancer^40^.

According to our results, the mean and SD of the age in the matched case and control group was 46.63±11.77 years, and in the unmatched control group, it was 49.61±8.39. Age was reported to be significantly associated with breast cancer. For every 1-year increase in a person’s age, the risk of breast cancer decreases by 2%, which can be caused by the identification of patients at a young age and the low age at which the disease is diagnosed. According to the statistics of the American Cancer Society in 2017, in the United States, women over the age of 70 years are at 3.9% (1 in 25), women in their 50s at 2.3% (1 in 43), and women in their 60s at 3.4% (1 in 29) risk of breast cancer in the next 10 years. Also, in older women, advanced stages of breast cancer are likely to be observed. At the same time, treatments are generally less effective, and compared to younger breast cancer patients, they are less likely to undergo standard or aggressive treatments^41–43^.

In the present study, the age of marriage and the age of the first pregnancy showed a significant relationship with the incidence of breast cancer in such a way that with an increase of 1-year in the age of marriage, the risk of developing breast cancer decreases by 17%, which can indicate the marriage of the studied patients at a young age, and with an increase of 1-year in the age of the first pregnancy, the risk of breast cancer increases by 26%. In 2019, in a study in Mazandaran, Mousavizadeh *et al*. showed that the chance of getting breast cancer in people with the age of first pregnancy over 22 years was 76.2 times that of people with the age of first pregnancy 22 years or less. According to the results of this study, the age of first pregnancy is one of the main risk factors with high strength for breast cancer in the region^44^. Marzbani *et al*. conducted a study on breast cancer in women under 50 years of age in Kermanshah in 2014. It was found that the age of the first delivery, between 18 and 35 years old, and the history of a benign mass in the breast are important risk factors for developing breast cancer in women under the age of 50, education about breast self-examination and the importance of periodic examinations in women can play an important role in preventing breast cancer^45^.

According to the results of the present study, the risk of breast cancer is slightly reduced by having physical activity, although this difference was not significant. There are various other factors that only slightly increase the risk of breast cancer that we may be able to control. For example, living a healthy lifestyle may help reduce the risk of breast cancer. Physical activity is associated with reducing the risk of breast cancer^33^.

A family history of breast and ovarian cancer was reported in the matched control group (*P*=0.368) and the unmatched control group (*P*=0.245). Based on this, no significant relationship was observed between the two groups in terms of family history and breast cancer, which could be due to the sensitivity created among the patient’s family members towards breast cancer and gaining awareness of the risk factors affecting the occurrence of the disease and healthy lifestyle choices. Also, service providers in health centers prioritize the follow-up of health care related to the prevention and screening of breast cancer among those around the affected, and in healthy people over 40 years of age who have a family history of cancer, annual screening mammography is recommended in addition to monthly self-examination. However, in other studies, the role of this factor in the occurrence of breast cancer has been reported. One of the most important risk factors for breast cancer is having a positive family history in first and second-degree relatives. There is a lot of evidence regarding this fact^46–50^.

According to the results of the study, with the increase in BMI, the incidence of breast cancer was higher; however, this rate was not significant. Our results were in line with the studies of Jun-J *et al*. conducted in China in 2010 to determine elderly breast cancer pathology features and treatment sensitivity; the results showed the age of menstruation and BMI were similar in both healthy and sick groups and had no significant relationship with breast cancer^51^. However, in other studies, a significant relationship between breast cancer and obesity was observed. Obesity in postmenopausal women is associated with an increased risk of breast cancer. Excess fat increases the plasma level of estrogen and contributes to the development of breast cancer^52^. In the analysis of parameters such as age and BMI, a direct and effective relationship with the incidence of breast cancer has recently been observed^53^.

According to the results of the present study, with the increase in blood pressure, the rate of breast cancer also increased, so with an increase of 10 mm of mercury in blood pressure, the rate of cancer increases by 7%. A study by Abbas Tabar and colleagues in Shiraz demonstrated that there was a significant relationship between the occurrence of breast cancer in women with diabetes and high blood pressure^54^. A study by Sun and his colleagues in 2015 in Taiwan showed that patients with high blood pressure are at higher risk for all types of cancer, and sex, age, and duration of high blood pressure have a significant relationship with cancer^55^. A study by Peters and colleagues in 2000 in the Netherlands showed that women with high blood pressure had a 23% increased risk of breast cancer^56–60^. A study by Chuang and his colleagues in Taiwan in 2015 showed that there was a significant relationship between obesity, high blood pressure, high blood cholesterol, and the occurrence of breast cancer (47–49). In a systematic review in 2017, Hun *et al*. found a positive association between high blood pressure and breast cancer incidence in Asian women^61–63^.

In the present study, people with diabetes were at a higher risk of developing breast cancer. Studies have shown that insulin and glucose are associated with an increased risk of breast cancer^64^. Also, persistent hyperglycemia, hyperinsulinemia, and insulin resistance increase insulin-like growth hormone^46^, which is associated with an increased risk of cancer initiation and progression^47^.

In the present study, it was found that high cholesterol increases the risk of breast cancer. That is the case with the studies by Jalilian *et al*. and Fadaeipour *et al*., too; one of the important and dangerous factors in increasing the risk of breast cancer is the patient’s lipid profile. Comorbidities, such as diabetes, hypertension, along with cancer, can affect survival outcomes^48,49^.

In the present study, no significant relationship was observed between vitamin D intake and increased risk of breast cancer. But in 2018, Tan *et al*. conducted a study in Malaysia titled ‘A case–control study of breast cancer factors in 7663 women in Malaysia’, and the results of the study showed that the duration of breastfeeding, frequency of breastfeeding, higher consumption of soy and plant products and a higher level of physical activity was associated with a lower risk of breast cancer^65^. Vitamin D performs various immunogenic and antiproliferative activities by binding to vitamin D receptors located in the nucleus of breast cells, among other body tissues^51^. This is why low vitamin D levels may lead to cancer by disrupting cell proliferation, differentiation, and apoptosis^52^. It has been found that people who are more exposed to sunlight, consume more vitamin D, or have a higher serum level of vitamin D have a decreased frequency of breast, colon, and prostate cancer^53^.

There was no significant relationship between the breastfeeding period and the risk of breast cancer in our study. Some studies have reported no protective effect for breastfeeding^12,66^. Alsolami and his colleagues in Saudi Arabia in 2019 reported that no effect of diabetes, high blood pressure, and duration of breastfeeding was observed on the occurrence of breast cancer. Tan *et al*.’s study was conducted in Malaysia in 2018 to investigate breast cancer risk factors and change risk factors; the results of the study showed that the duration of breastfeeding and the frequency of breastfeeding were associated with a lower risk of breast cancer. Chinese women participating in the study had the lowest frequency of breastfeeding, the lowest duration of breastfeeding period, the lowest frequency of childbirth, and the highest age of first pregnancy^65^.

### Study limitations

This research’s statistical population is the files registered in the integrated system of the Ministry of Health and Medical Education from the beginning of 2016 to the end of 2021.
The statistical population of this research is women aged 30–69.
Participants did not report the status of using tobacco, drugs, and alcoholic beverages in any group, which may be due to geographical conditions, genetics, quality of life, nutrition, and failure to provide correct information to health workers due to fear of judgment, social factors, and concealment among others.
In the examination of the control groups, the history of benign cysts and breast sampling was not reported because the condition for entering the control group was a normal breast cancer screening, which includes the absence of abnormal appearance in the breast, the absence of lumps or cysts on physical examination, and a negative history of biopsy.

## Conclusion

The results of the univariate analysis showed that the age of first pregnancy, type of delivery, high blood cholesterol, high blood pressure, and diabetes have a significant relationship with breast cancer, and according to the results of multivariate analysis, the age of first pregnancy and type of delivery are predictors of the disease. Therefore, by performing breast cancer screening in women who are exposed to these risk factors, early diagnosis of the disease and increasing the speed of their treatment can be greatly helped.

## Ethical approval

After the proposal was approved by the university's research council and after obtaining the ethics code number (IR.IAU.CHALUS.REC.1400.053), we received a letter of introduction from Mazandaran Islamic Azad University. We presented it to the Ardabil University of Medical Sciences, and in coordination with senior officials of Ardabil University of Medical Sciences, a permit was obtained to access the information of women aged 30–69.

## Consent

To observe ethical considerations, in this study, issues such as obtaining an ethics code with the number IR.IAU.CHALUS.REC.1400. 053, maintaining confidentiality of information at all stages were respected. Given that the data collected was of a secondary nature and did not involve human sampling, the requirement for obtaining informed consent did not apply. This determination was confirmed by the Ethics Committee of Islamic Azad University Mazandaran. The data were received from the electronic systems of the Iranian Ministry of Health (Ardabil University of Medical Sciences) from http://sib.arums.ac.ir/ after correspondence and receiving the ethics code.

## Sources of funding

There was no financial support in the design of the study, data collection, analysis, and interpretation of the results and writing of this article.

## Author contribution

M.-A.J.: investigation, project administration, resources, supervision, writing – original draft; B.G.: investigation, resources, software, writing – review and editing; S.A.S.: investigation, writing – review and editing; M.N.: data curation, formal analysis, and writing – review and editing; H.-A.N.: formal analysis and writing – original draft; S.N.H.: data curation, formal analysis, and visualization; J.Y.C.: data curation, formal analysis, methodology, software, validation, and visualization; G.M.: conceptualization, data curation, formal analysis, investigation, supervision, writing – original draft, and writing – review and editing.

## Conflicts of interest disclosure

The authors have declared no conflicts of interest.

## Research registration unique identifying number (UIN)

Approval and registration of the proposal with project code 162314609 in the Mazandaran University Research Council and then obtaining the ethics code number (IR.IAU.CHALUS.REC.1400.053).

## Guarantor

Mohammad-Ali Jahani(Ph.D) Social Determinants of Health Research Center, Health Research Institute, Babol University of Medical Sciences, Babol, Iran.

Ghahraman Mahmoudi(Ph.D) Hospital Administration Research Center, Sari Branch, Islamic Azad University, Sari, Iran.

## Data availability statement

The datasets used and/or analyzed during the current study are available from the corresponding author on reasonable request.

## Provenance and peer review

our paper was not invited.
